# Alloying Gold

**Published:** 1857-06

**Authors:** G. Watt


### ALLOYING GOLD.

BY G. WATT.

Young dentists, and perhaps *some* of the old ones, are often puzzled in regard to one of the modes of expression used in designating the quality or fineness of gold alloys. The term "carat" is often a stumbling-block, and is frequently used indefinitely, sometimes, indeed, without any meaning at all. The term is a relative one. The quantity of gold under consideration is supposed to be divided into 24 parts, and each of these parts may be considered a carat. Hence, pure gold is said to be 24 carats fine, or simply "fine gold." If one of the 24 parts be a baser metal than gold, the alloyed mass is said to be 23 carat gold; if 6 of the parts are base metal, it s 18 carat gold; if one-half be base metal, it is 12 carats, etc. By observing this, the whole thing is perfectly plain. For example, if we speak of 19 carat gold, we simply mean that every 19 parts of pure gold it contains are united with five parts of silver, copper, or some other metal.

Much of the gold plate used by dentists is alloyed by guess, and more still, perhaps, by arbitrary rules or recipes, without any reference to, or knowledge of the quality of plate produced. This, to say the least of it, is not satisfactory to the individual operator, nor flattering to the profession. It is annoying to any man of spirit to work without a proper knowledge of that with which he works; and when this can be so readily obtained in the present case, there is but little sympathy due to those who permit themselves to be long thus annoyed.

In compliance with the request of some of the younger members of the profession, we will give a few simple rules, which we find convenient in our own practice. We are not aware that there is any thing new or peculiar about them, nor will we try to be very technical or concise in the statement of them. We wish the principles on which the rules are founded to be understood, and then they can not be forgotten, while, if these are not understood, they will not be remembered. The rules will be more readily understood by bearing in mind that 24 is the number of parts into which any quantity of gold under consideration is supposed to be divided.

Let us suppose, then, that it is desired,

1. *To ascertain the carat of any given alloy.*

The proportion may be expressed as follows:

As the weight of the alloyed mass is to the weight of gold it contains, so is 24 to the standard sought. Take, for example, Harris's No. 3 gold solder:

| | | |
|---|---|---|
| Pure gold, | 6 | parts. |
| " silver, | 2 | " |
| " copper, | 1 | " |
| Total | 9 | " |

The proportion would be expressed thus—9 : 6 :: 24 : 16.

From this any one can deduce the following

RULE.—Multiply 24 by the weight of gold in the alloyed mass, and divide the product by the weight of the mass; the quotient is the carat sought.

In the above example, 24 multiplied by 6, the quantity of gold, gives 144, which, divided by 9, the weight of the whole mass, gives 16. Hence, an alloy prepared as above, is 16 carats fine.

As another example, under the same rule, take Harris's No. 1 solder:

| | | |
|---|---|---|
| 22 carat gold, | 48 | parts. |
| silver, | 16 | " |
| copper, | 12 | " |
| Total.................................... | 76 | " |

Now, as the gold used is but 22 carats fine, one-twelfth of it is alloy. The one-twelfth of 48 is 4, which, subtracted from 48, leaves 44. The statement then is:

$$76 : 44 :: 24 : 13.9.$$

This solder, therefore, falls a fraction below 14 carats.

2. *To reduce gold to a required carat.*

The proportion may be expressed as follows:

As the required carat is to 24, so is the weight of gold used to the weight of the alloyed mass when reduced. The weight of the gold subtracted from this, gives the quantity of alloy to be added.

For example, reduce 6 ounces of pure gold to 16 carats. The statement is expressed thus:

$$16 : 24 :: 6 : 9.$$

Six subtracted from 9 leaves 3, which is the quantity of alloy to be added. From this is deducted the following

RULE.—Multiply 24 by the weight of pure gold used, and divide the product by the required carat. The quotient is the weight of the mass when reduced, from which subtract the weight of the gold used, and the remainder is the weight of alloy to be added.

As another example under the same rule, reduce 1 penny weight of 22 carat gold to 18 carats.

As the gold is only 22 carats fine, one-twelfth of it is already alloy. The one penny-weight, therefore, contains but 22 grs. of pure gold. The statement is, therefore, thus expressed:

18 : 24 : : 22 : $29\frac{1}{3}$. Twenty-four subtracted from $29\frac{1}{3}$ leaves $5\frac{1}{3}$. Therefore, each penny-weight of 22 carat gold requires $5\frac{1}{3}$ grains of alloy to reduce it to 18 carats.

3. *To reduce gold from a lower to a higher carat.*

This may be done by adding pure gold, or a gold alloy finer than that required. The principle of the rule may be set forth in the following general expression:

As the alloy in the required carat is to the alloy in the given carat, so is the weight of the alloyed gold used to the weight of the reduced alloy required. This principle may be practically applied by the following

RULE.—Multiply the weight of the alloyed gold used by the number representing the proportion of alloy in the given carat, and divide the product by that representing the proportion of alloy in the required carat; the quotient is the weight of the mass when reduced to the required carat by adding fine gold.

To illustrate this rule, take the following examples:

Reduce 1 penny-weight of 16 carat gold to 18 carats.

The numbers representing the proportions of alloy in this example are found by respectively subtracting 18 and 16 from 24. The statement is, therefore,

$$6 : 8 : : 1 : 1\tfrac{1}{3},$$

from which it follows that to reduce one penny-weight of 16 carat gold to 18 carats, there must be one-third of a penny-weight of pure gold added to it.

But, suppose that, instead of pure gold, we wish to effect the change by adding 22 carat gold. The numbers then respectively representing the proportions of alloy would be found by subtracting, in the above example, 16 and 18 from 22, and the statement would be,

$$4 : 6 : : 1 : 1\tfrac{1}{2}.$$

It follows, then, that to each penny-weight of 16 carat gold, a half-penny-weight of 22 carat gold must be added, to bring it to 18 carats.

By the above rules we think the student will be able, in all cases, to calculate the quality or fineness of his gold, and to effect any reduction, whether ascending or descending, which he may desire. This article is written expressly for dental students and the younger class of practitioners. Many of the older members will, doubtless, laugh at it, and regard it as very *simple* (?) but they should remember that though it may be a long time since they were young, yet it is not long since we were, and, therefore, not long since we very much desired just the simple information contained in the above.

